# A panorama of radial nerve pathologies- an imaging diagnosis: a step ahead

**DOI:** 10.1007/s13244-018-0662-x

**Published:** 2018-11-05

**Authors:** Aakanksha Agarwal, Abhishek Chandra, Usha Jaipal, Narender Saini

**Affiliations:** 1 Department of Radiodiagnosis and Modern Imaging, SMS Medical College and Attached Hospitals, Jaipur, Rajasthan India; 2 Department of Orthopaedics, SMS Medical College and Attached Hospitals, Jaipur, Rajasthan India

**Keywords:** High-resolution ultrasound diagnosis of radial neuropathy, Radial nerve palsy, Morphological assessment of radial nerve

## Abstract

**Abstract:**

The radial nerve has a long and tortuous course in the upper limb. Injury to the nerve can occur due to a multitude of causes at many potential sites along its course. The most common site of involvement is in the proximal forearm affecting the posterior interosseous branch while the main branch of the radial nerve is injured in fractures of the humeral shaft. Signs and symptoms of radial neuropathy depend upon the site of injury. Injury to the nerve distal to innervation of triceps brachii results in loss of extensor function with sparing of function of the triceps resulting in the characteristic ‘wrist drop’. Injury in the mid-arm is associated with loss of sensation in the dorsolateral aspect of the hand, the dorsal aspect of the radial three-and-a-half digits and in the first web space. Involvement of only the posterior interosseous nerve (PIN) results in weakness of the wrist and digit extensors. Diagnosis relies on clinical examination, electrodiagnostic studies and imaging findings. Plain radiographs are used to identify fracture sites, callus or tumours as cause of compression. Technological advances in ultrasonography have allowed direct visualisation of the involved nerve with assessment of the exact site, extent and type of injury. It yields unmatched information about anatomical details of the nerve. MR imaging adds to soft-tissue details and helps in characterising the lesion. This pictorial review aims to illustrate a wide spectrum of causes of radial neuropathy and emphasises the importance of imaging modalities in diagnosis of neuropathies.

**Teaching Points:**

• *Radial nerve injuries are assessed by clinical examination and diagnosed using electrodiagnostic and imaging studies*.

• *Knowledge of anatomical relations and course of the nerve is necessary to identify the nerve at pre-determined anatomical locations.*

• *Altered echogenicity and signal intensity, discontinuity of the nerve, focal thickening and cause of compression can be assessed by imaging modalities.*

• *MR imaging helps in confirmation of the ultrasound findings, differentiating similar appearing lesions and provides additional soft-tissue details.*

## Introduction

Radial nerve pathologies include compressive syndromes, entrapment neuropathy, focal intrinsic lesions and peripheral nerve sheath tumours. The radial nerve has a long and tortuous course in the upper limb and lies in close proximity to the bone in the spiral groove, making it susceptible to injuries. The prevalence of radial nerve palsy following fracture of the humerus shaft is 11.8% [1], while incidence of iatrogenic injuries is approximately 4.2% [2]. Clinically, radial neuropathy presents as wrist drop with or without sensory loss along the posterior surface of arm, forearm, thenar eminence and dorsal aspect of radial three and a half digits, depending upon the site of injury. Conventionally, neuropathies have been diagnosed on the basis of clinical examination, Tinel’s sign and electrodiagnostic (nerve conduction velocity and electromyography) findings which provide information about the nerve involved and possible site of injury.

Introduction of high-frequency ultrasound probe has made direct visualisation of peripheral nerves possible, thus providing anatomical details about the nerve. Ultrasonographic findings of peripheral nerves were first reviewed by Fornage in 1988 [3]. Since then, technological advances like increased frequency and variable sizes of footprints of linear transducers have escalated the use of ultrasound in peripheral nerve pathologies. Exact site, extent and type of involvement, local cause of neuropathy, continuity of nerve and architectural distortion can be determined which helps the clinician in appropriate decision making regarding the need for urgent surgical intervention. Ultrasound is quick, less expensive, has no contraindications and provides detailed imaging of the entire length of the nerve. MR imaging adds to soft-tissue details, change in muscles supplied by the affected nerve and helps in characterising the nerve lesion. Despite these advantages, imaging remains underutilised in cases of peripheral neuropathy. With this pictorial review, we aim to put forward a panoramic view of various causes of radial nerve palsy as diagnosed using imaging modalities.

Various previous publications have mentioned methods to identify the nerve in specific anatomical locations and various peripheral nerve pathologies [3–7]. Extensive literature search, however, did not reveal any publication focussed on imaging findings in such a wide spectrum of radial nerve pathologies.

## Anatomical details [6]

The radial nerve originates from the ventral rami of C5 – T1 and lies in the posterior cord of the brachial plexus with the axillary artery as its anterior relation. Within the axilla, it has three branches: sensory posterior cutaneous nerve of the arm and motor branch to the long and medial head of the triceps. It passes anterior to the subscapularis, latissimus dorsi and teres major. This is the first possible site of compression. An anomalous muscle, the accessory subscapularis-teres-latissimus is reported in literature to be another cause of compression of the radial nerve at this level [8]. Another potential cause of compression, as described by Spinner [9] is penetration of the nerve directly by the subscapular artery more distally in the axilla, forming a neural loop.

**Fig. 1:**
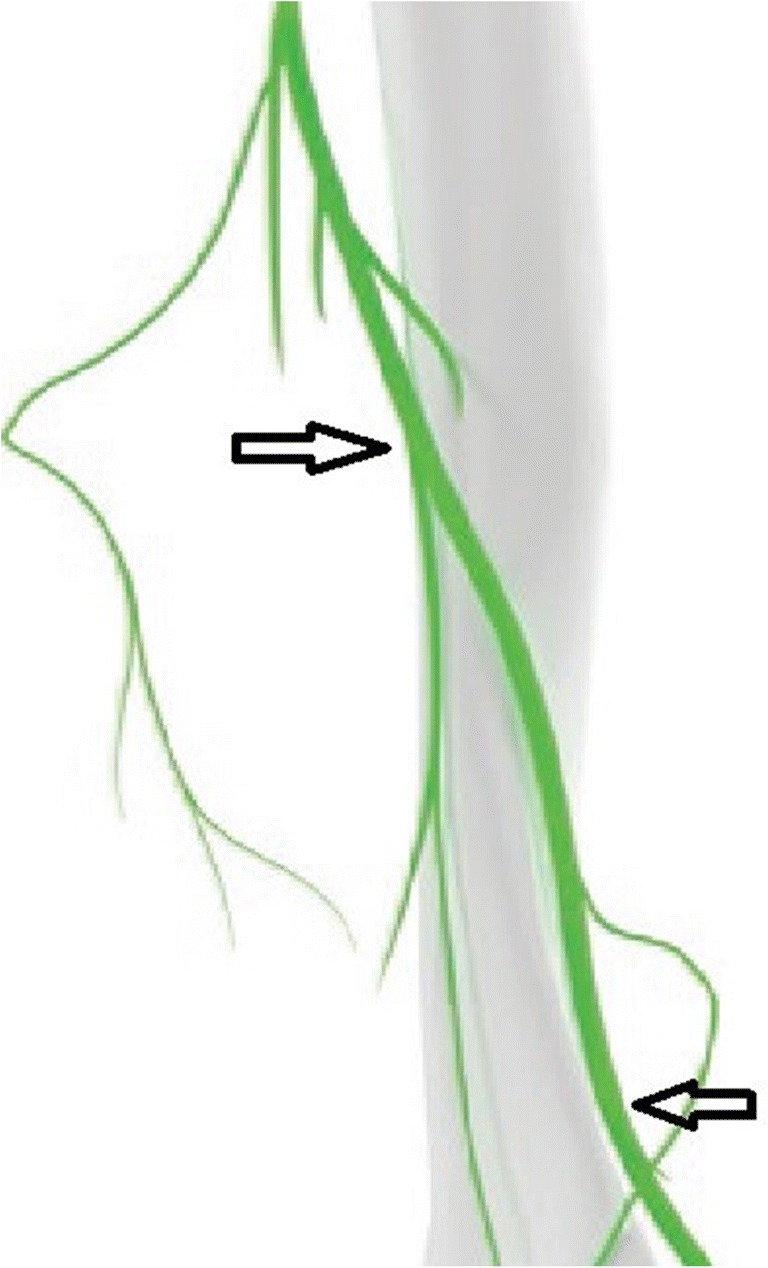
Black arrows denote the site where the radial nerve is easily identified in its short axis: in the spiral groove and distally between the brachialis and brachioradialis. [13]

**Fig. 2:**
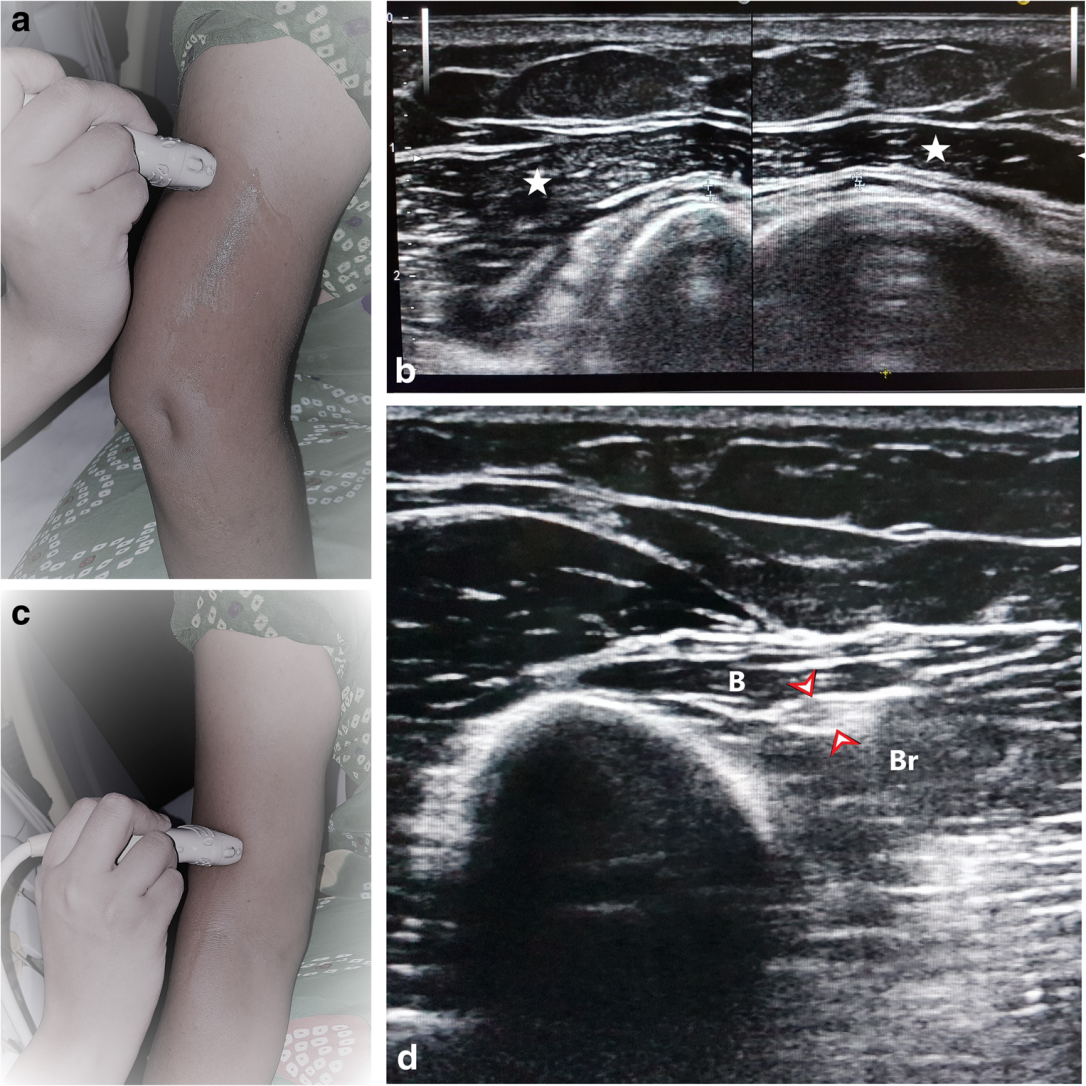
**a** Probe position for localising the nerve in the spiral groove. The patient’s arm is kept extended, internally rotated and the posterolateral aspect of the arm is scanned. **b** Crossheads denote the normal honeycomb appearance of radial nerve in the spiral groove between lateral and medial heads of the triceps (stars) **c** Probe position for localising the nerve between the brachialis and brachioradialis. The lateral part of arm is to be scanned with the arm slightly flexed at the elbow and internally rotated. **d** Arrowheads denote the normal honeycomb appearance of the radial nerve in the distal arm lying between the brachialis (B) and brachioradialis (Br)

It enters the posterior compartment of the arm by travelling through the triangular interval bounded by the teres major superiorly, the long head of the triceps medially and the lateral head of the triceps laterally. The radial nerve then exits the axilla, courses through the lateral head of triceps brachii and winds around the spiral groove accompanied by the profunda brachii artery. Lorem et al. [10] described this as another possible site of entrapment. Familial radial nerve entrapment syndrome [11] also occurs secondary to compression at the lateral head of the triceps. Intermittent compression of the nerve may also occur secondary to genetic defect in Schwann cell myelin metabolism [12]. The radial nerve gives off the following branches in the upper arm - the inferior lateral cutaneous nerve of the arm, posterior cutaneous nerve of the forearm and motor branches to the lateral head of triceps and anconeus. The nerve is in close proximity to the humerus in the spiral groove and is easily identified on ultrasound in its short axis overlying the humerus between the lateral and medial heads of the triceps. (Figs. 1, 2a and b). The nerve then courses from the posterior to anterior compartment of the arm by piercing the lateral intermuscular septum which is another possible site of entrapment.

Thereafter, it descends in the arm and comes to lie between the brachialis and brachioradialis giving motor supply to both the muscles in addition to the extensor carpi radialis longus (ECRL) and extensor carpi radialis brevis (ECRB). This too is a constant relation which can be identified on ultrasound when scanning the lateral aspect of the lower arm (Figs. 1, 2c and d). The nerve courses anterior to the lateral epicondyle into the forearm and divides into superficial and deep branches (posterior interosseous nerve, PIN). The PIN winds around the radial head, lying between two heads of the supinator muscle and then travels on the dorsal surface of the interosseous membrane. The superficial layer of the supinator muscle forms the arcade of Frohse which is the most common site of entrapment neuropathy causing the radial tunnel syndrome [14]. Two anomalous courses of the PIN have been reported in the literature by Woltman and Learmonth. First is passage of the nerve within the substance of the supinator, and the other involves a branch travelling superficial to the supinator brevis [15]. The origin of the radial tunnel is where the deep branch (lateral) courses over the radio-humeral joint. The tunnel ends at the distal edge of the superficial supinator where the PIN is formed. Radial tunnel syndrome can be caused by the arcade of Frohse, fibrous fascial bands coursing superficial to the nerve or the vascular leash of Henry [16] which is formed by the recurrent radial vessels. The PIN supplies the abductor pollicis longus, the extensor pollicis brevis, the extensor indicis proprius and the extensor pollicis longus.

The superficial branch courses medially, deep to the brachioradialis, lateral to the radial artery into the wrist. It pierces the lateral fascia to enter the anatomical snuffbox and gives sensory innervation to the dorsal surface of three and a half digits on the radial aspect. It also innervates the extensor digitorum, the extensor digiti minimi and the extensor carpi ulnaris muscles.

## Technical aspects of imaging

The normal peripheral nerve has a honeycomb appearance which is identified on imaging. The central spots are the nerve fascicles which are formed by bundles of nerve fibres surrounded by the perineurium. Each nerve fibre is also surrounded by an endoneurium which is not visible on imaging with the currently available resolution. The fascicles are in turn embedded and surrounded by the epineurium which forms the sheath of the peripheral nerve [17]. The dimensions of the radial nerve in a study by Husarik et al. [18] in 60 asymptomatic patients yielded the following results: At 2 cm proximal to the joint space, the dimensions of the radial nerve varied between 1.0 and 5.0 mm (median, 2.0 × 4.0 mm). After the radial nerve bifurcated into the superficial radial nerve and PIN, the diameters were smaller and varied between 0.8–2.0 mm (median, 1.1 × 1.7 mm) and 0.8–3.1 mm (median, 1.0 × 2.0 mm), respectively.

High-resolution ultrasound: A high-frequency linear probe is required for scanning peripheral nerves. We used a probe with frequency set at 14 Hz. The nerve should be identified in a cross section as it has a characteristic honeycomb appearance which helps in demarcating it from the surrounding soft tissue. After identifying the nerve in its short axis at pre-determined anatomical locations, it can be traced proximally and distally throughout its length. The nerve is more echogenic to the surrounding muscle and less echogenic to the surrounding tendon. The pathological segment of the nerve will show loss of the normal honeycomb structure, decreased echogenicity, discontinuity, focal enlargement or neuroma formation. Adjacent compressive lesions, muscle and soft-tissue oedema, and joint effusions can also be identified.

MR imaging: We used a 3-T MRI scanner with a dedicated protocol for visualisation of the radial nerve. T1, T2 fat-saturated and proton density (PD) fat-saturated sequences were acquired with a slice thickness of 3 mm and interslice gap of 0.3 mm. Gadolinium-based contrast was used for post-contrast images if required. Knowledge of the anatomical relations of the radial nerve is necessary to identify the nerve in its short axis. T1-weighted images allow clear visualisation of the nerve in its short axis as the peripheral rim of fat in the nerve sheath appears hyperintense while the fascicles appear as dots, hypointense to the surrounding muscles. Epineurial or perineurial fat remains bright on T2-weighted imaging (T2WI) which can mask pathological changes in the nerves. Fat suppression is thus needed for identification of pathological segment of nerve on T2 and PD sequences [19, 20]. Focal enlargement, hyperintensity and altered fascicular patterns are signs of pathology in the nerve. Nerve hyperintensity may be seen secondary to autoimmune or multifocal motor neuropathies and requires clinical co-relation. The denervated muscles begin to appear hyperintense on T2-weighted images 48 h after nerve injury. These changes are seen in axonotmetic and neurotmetic injuries. MRI may not clearly differentiate between disruption injuries and contusion of the nerve [21]. Muscle atrophy and persistent hyperintensity is seen in failure of regeneration [20]. Fatty infiltration is seen in chronic denervation. Orthopaedic implants may limit the use of MRI due to susceptibility artefacts.

## Clinical presentation and diagnosis

Patients with radial neuropathy can present either with motor dysfunction or sensory symptoms only. Pain over the lateral aspect of the arm extending up to the wrist is the most common complaint in cases of radial tunnel syndrome [22]. Injury to the radial nerve high up in the arm can result in complete loss of extensor function while injuries distal to the supply of triceps brachii spares its function. Fracture of the humerus shaft can result in wrist drop as injury to the nerve usually occurs in the spiral groove or in the mid-arm [23]. The patients will also have loss of sensation over the lateral aspect of arm, forearm, thenar eminence and radial three and a half digits.

Compression of the superficial branch causes paralysis of the extensor carpi ulnaris, extensor digiti quinti and extensor digitorum communis, while compression of the PIN causes paralysis of the abductor pollicis longus, extensor pollicis brevis, extensor pollicis longus and extensor indicis proprius. Function of the extensor carpi radialis longus is spared as the fibres arise more proximally from the main branch of the radial nerve. This results in finger drop with deviation of the wrist to the radial aspect which is the classical presentation in cases of PIN palsy. Wartenburg syndrome is entrapment of the superficial branch at the distal radius and presents with pain and numbness in the posterior part of the thumb [24].

Patients with peripheral nerve sheath tumours may present with visible swelling with no motor or sensory deficit.

Clinical examination and electrodiagnostic studies (nerve conduction velocity [NCV] and electromyography [EMG]) have been the mainstays of diagnosis in neuropathies as they provide essential information about the type of dysfunction and help in clinical monitoring. EMG may not be positive for 3–6 weeks following injury. Tinel’s sign helps in assessing the possible site of injury and helps in assessment of improvement over time. Nerve injuries were classified by Seddon in 1943 and expanded by Sunderland in 1951. Mackinnon and Dellon in 1992 added grade VI injury to Sunderland’s grading scheme and defined it as a mixed type of injury which denotes various types of injuries across the cross section of the nerve [25–27]. Table 1 illustrates this classification along with imaging findings in different types of nerve injuries.

**Table 1:** Co-relation between Seddon’s & Sunderland’s classification of nerve injury with imaging findings

Seddon	Sunderland	Description	MRI	Ultrasound
Neuropraxia	I	Conduction block	T2 hyperintensity	Decreased nerve echogenicity (hypoechoic)
Axonotmesis	II	Discontinuity of the axon with Wallerian degeneration	T2 hyperintensity with increased size. Hyperintensity in muscles due to denervation.	Decreased echogenicity and increased calibre of the nerve
	III	Scarring of the endoneurium	Endoneurium can not be delineated with current MR technique. T2 hyperintensity with increased size. Hyperintensity in muscles due to denervation.	Focal decrease in echogenicity with increase in calibre with change in echotexture of the affected muscles.
	IV	Neuroma in continuity with formation of a scar which blocks nerve regeneration	T1 hypointense, T2 hyperintense focal enlargement with loss of fascicular pattern. Hyperintensity in muscles due to denervation.	Hypoechoic fusiform lesion in continuity with the nerve with loss of fascicular architecture with altered echogenicity of denervated muscles.
Neurotmesis	V	Rupture of the nerve	End neuroma formation at proximal end with denervation changes in muscle	Hypoechoic neuroma at proximal end with local soft-tissue oedema and denervation changes in muscle.
	Mackinnon and Dellon type VI	Mixed injury	Variable findings with nerve heterogeneity and muscle denervation changes	Hypoechoic enlarged with mixed findings of scarring, discontinuity or neuroma formation.

A plain radiograph shows the fracture site in cases of post-traumatic radial nerve palsy. Callus formation and soft-tissue swellings can also be noted on plain radiographs. It may also demonstrate a fat stripe of lipoma or a bony lesion in proximity to the course of the nerve.

High-resolution ultrasound and MRI help in direct visualisation of the nerve with anatomical delineation of the pathological segment. These also provide essential information about the surrounding soft tissue and the muscles innervated by the injured nerve. Imaging studies help in better clinical decision making, need for operative interference, time of intervention and possible complications.

## Aetiology of radial nerve pathology [28]

Causes of radial nerve palsy are manifold with fracture of the humerus shaft being the commonest association. It can occur due to entrapment of the nerve at anatomical sites, entrapment within scar tissue, pressure by tourniquet, under-arm crutches, healing callus, etc. Iatrogenic radial nerve palsy is another common cause of injury which can occur after fracture reduction or after operative management of fracture of the humerus or radius. Elbow surgeries like arthroplasty, radial head fracture repair, synovectomy can cause PIN palsy. Penetrating injuries like gun shot injuries, animal bites, sharp objects, and intramuscular injections can cause direct insult to the nerve. Thermal and burn injuries are another cause of radial nerve palsy which may also include other nerves in the region of the burn. Systemic demyelinating diseases, lead poisoning or neurotoxic drugs may affect the radial nerve along with other peripheral nerves.

Tumour and tumour-like lesions involving the radial nerve include eripheral nerve sheath tumours (PNSTs), fibrolipomatous hypertrophy, intraneural lipomas, intraneural ganglion, true neuromas and pseudo neuromas [29].

## Imaging findings in a wide spectrum of radial nerve pathology

**Fig. 3:**
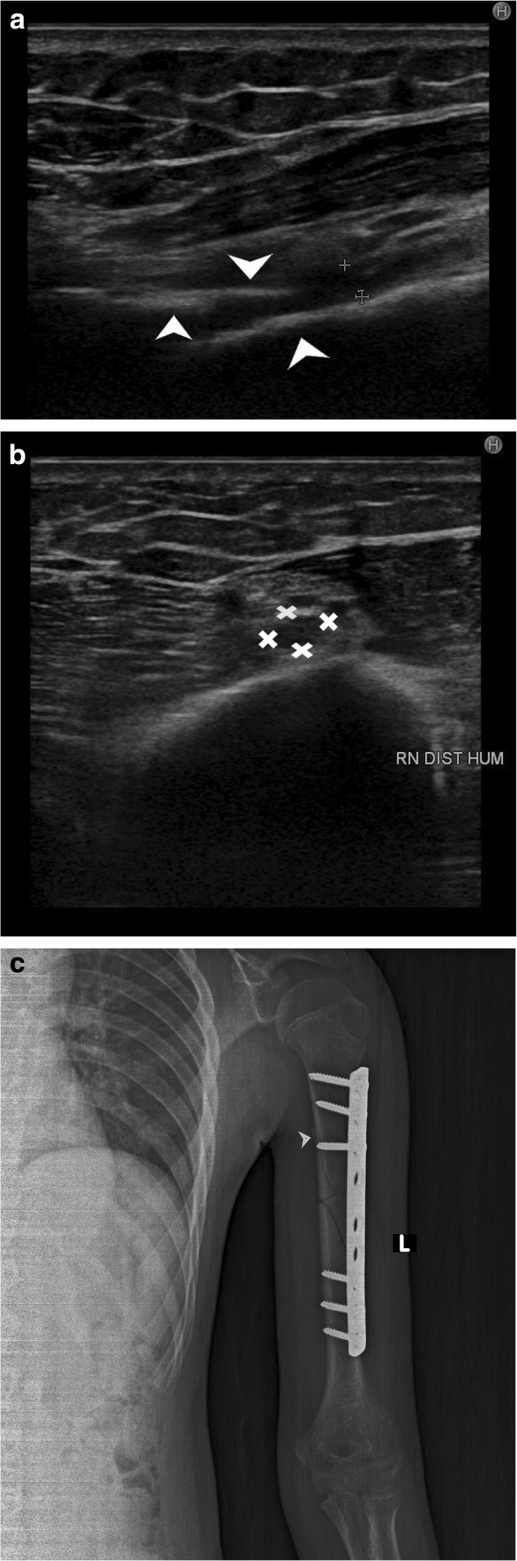
**a** Hypoechoic radial nerve is seen in the longitudinal plane (between crossheads) entrapped between two edges of the fracture segment (arrowhead). **b** Hypoechoic radial nerve, crossheads, in its short axis just distal to the site of entrapment. **c** Plain radiograph after open reduction and internal fixation in a patient with fracture of the humerus shaft (arrowhead). The nerve was impinged within the fracture segment in the spiral groove

**Fig. 4:**
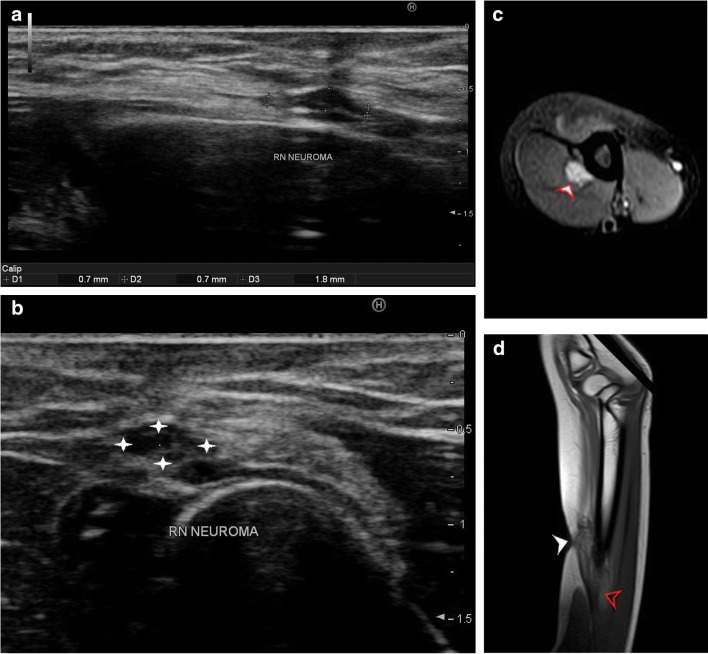
**a** Focal fusiform thickening of radial nerve is noted (D3 marker) with normal calibre proximally and distally (D1 and D2 markers). The nerve is hypoechoic at the site of enlargement of the nerve. This anatomical abnormality was noted just deep to the scar at the site of bite on the skin. **b** Short axis view of focal thickening suggestive of radial nerve neuroma (between cross marks) seen distal to the spiral groove. The nerve is seen lying between the brachialis and brachioradialis. **c** Axial PD fat saturated sequence of the same patient showing a well-defined ovoid hyperintense lesion (black arrowhead) in continuity with the radial nerve. As the lesion was noted distal to the spiral groove, after supply to the triceps, there is no signal intensity change in the triceps muscle signifying normal innervation to the muscle. **d** Sagittal PD sequence showing a fusiform hyperintense lesion (black arrowhead) corresponding with the ultrasound findings. The scar site on the skin can also be noted (white arrowhead)

**Fig. 5:**
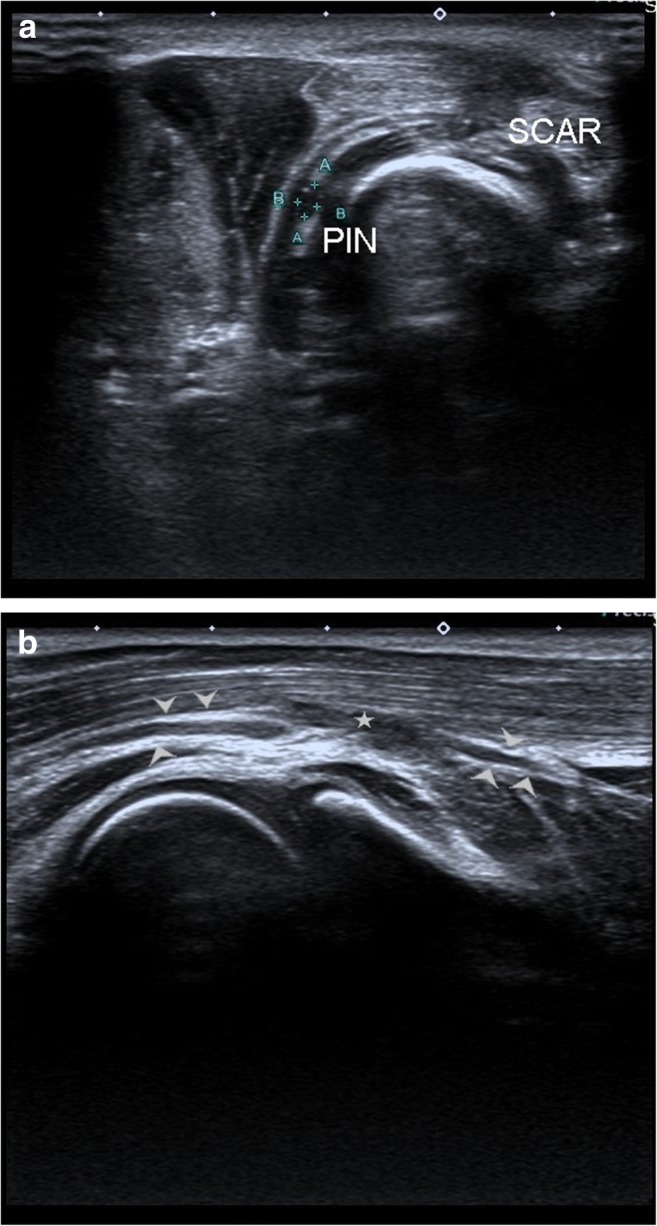
**a** High-resolution ultrasound image of a patient who had history of penetrating injury distal to the elbow. SCAR denotes the hypoechoic scar tissue formed within soft tissue at the site of injury. The posterior interosseous nerve (PIN) is normal at the level of local scar. **b** Image shows discontinuity in posterior interosseous nerve in the forearm with formation of hypoechoic scar tissue (stars), distal to the site of scar on skin. The nerve (arrowheads) is of normal calibre proximally and distally

**Fig. 6:**
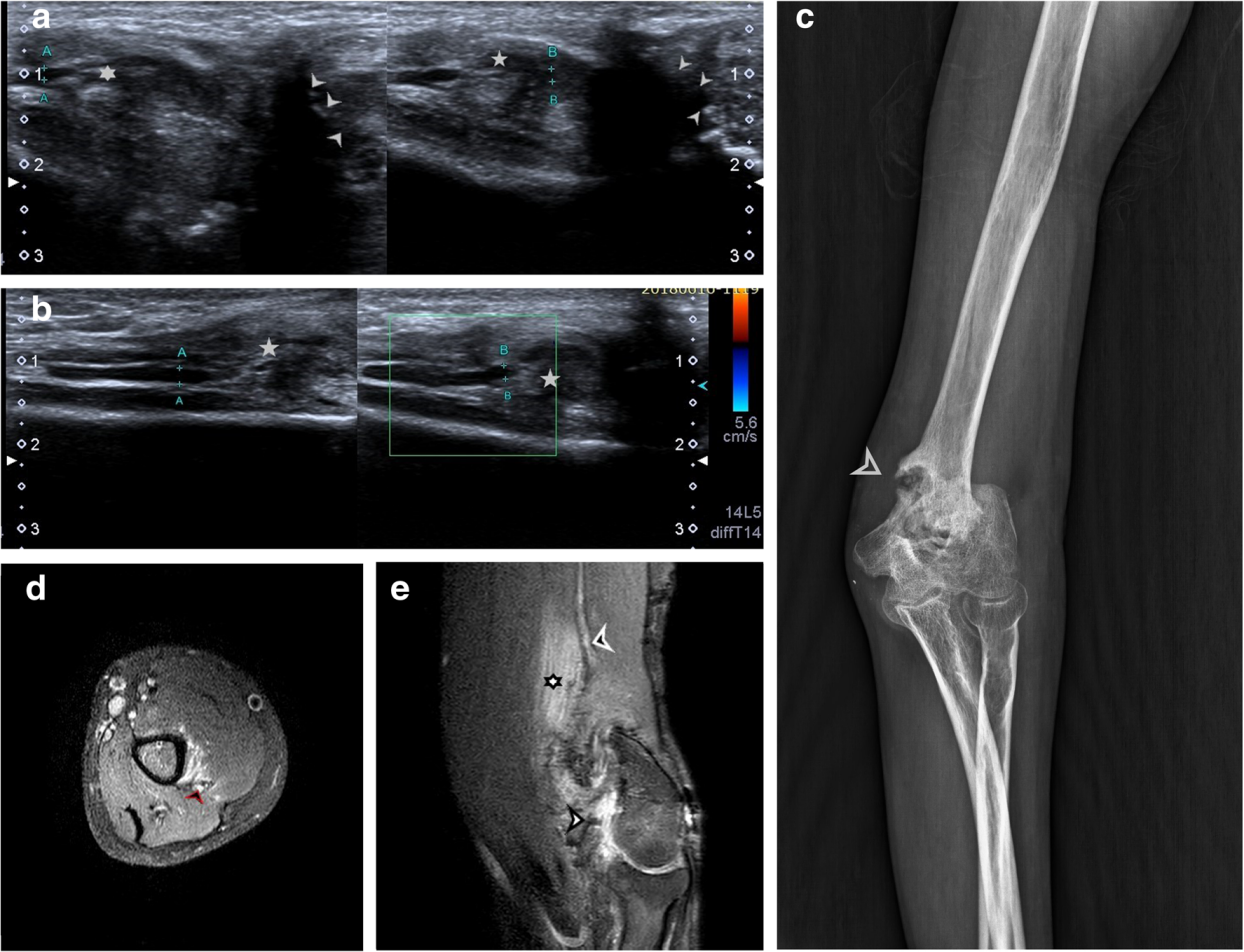
**a** Hypoechoic radial nerve (point A and B) is seen entrapped within mixed echogenic chronic inflammatory soft tissue (5 point and 6 point star). Malunited fracture of humerus can be seen adjacent to it (arrowheads). **b** Long axis view of hypoechoic radial nerve (point A and B) seen entering into the chronic inflammatory tissue. There was no evidence of increased vascularity of the nerve. **c** Plain radiograph showing malunited, displaced fragment of supracondylar fracture of humerus. **d** Axial PD sequence showing hyperintense radial nerve (black arrowhead) with surrounding hyperintense muscle oedema. **e** Sagittal PD sequence showing heterogeneously hyperintense area of chronic inflammation in region of malunited supracondylar fracture of humerus (white arrowhead). The radial nerve is enlarged and hyperintense (black arrowhead) and is not visualised within the area of chronic inflammation and surrounding muscle oedema (6 point star) due to similar signal intensities

**Fig. 7:**
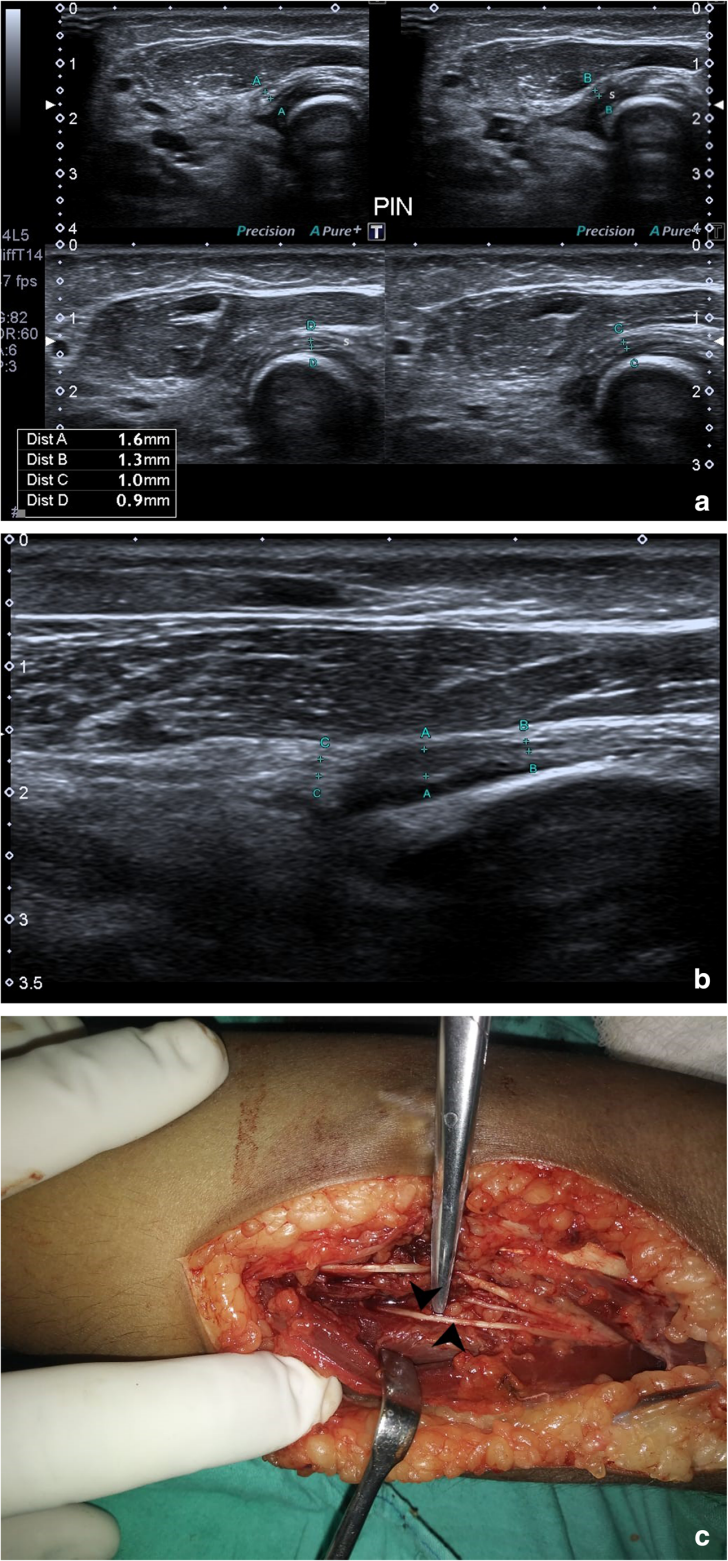
**a** Normal posterior interosseous nerve can be seen winding around the radial head, passing between two heads of the supinator (s) in to the forearm. The nerve is of normal calibre and echogenicity (A,B,C and D). **b** Just distal to 7a, focal increase in calibre and hypo-echogenicity (marker A) is noted in PIN. **c** Intraoperative picture of PIN (arrowhead) after release of nerve from surrounding local scar tissue

**Fig. 8:**
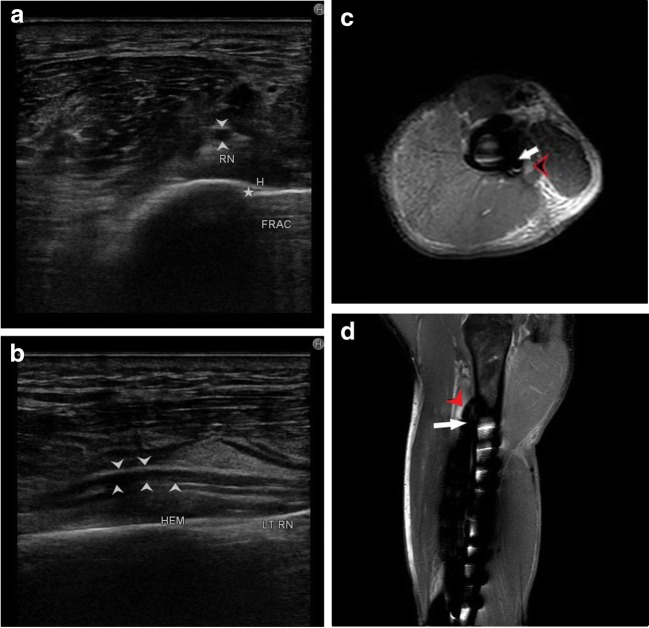
**a** Displacement of radial nerve (arrowhead) is seen due to a local hematoma (H) formed following traumatic fracture (asterisk) of humerus shaft. **b** Long axis view of the left radial nerve (arrowhead) showing displacement by local hematoma (HEM). **c**: Axial PD sequence showing hyperintense radial nerve (black arrowhead) displaced by underlying acute hematoma (white arrow) in a post-operative case of fracture of the humerus shaft. **d** Coronal PD sequence showing hyperintense radial nerve (black arrowhead) with underlying hematoma in acute stage. Also noted are screws of the medullary nail implanted for fixation of the fracture of the humerus shaft

**Fig. 9:**
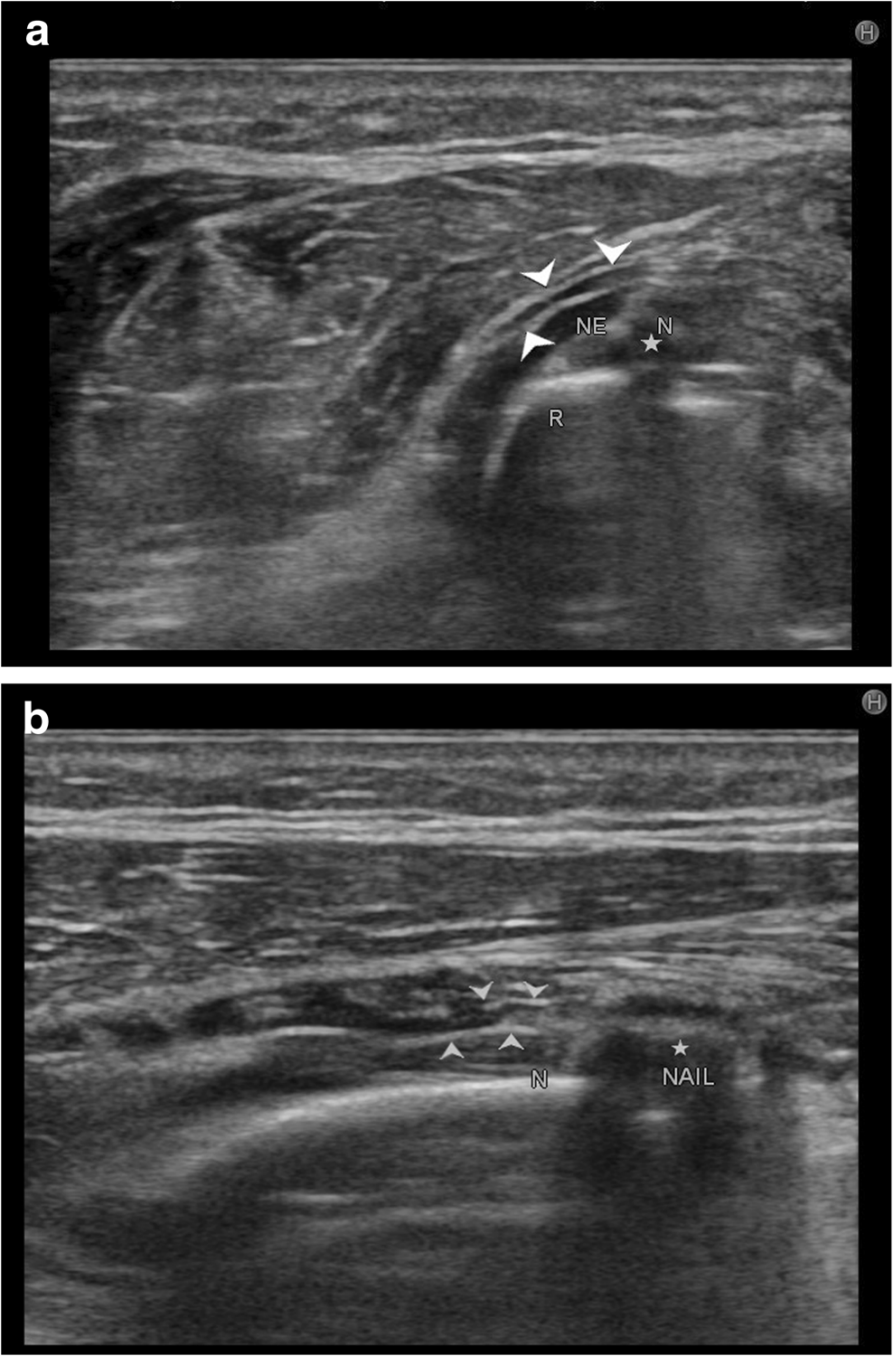
**a** Arrowheads show posterior interosseous nerve (NE) being impinged by nail (N, star) following internal fixation in a case of fracture of both bones of forearm. **b** Arrowheads show posterior interosseous nerve getting impinged by implant (star). The nerve has a wavy contour but normal calibre. The patient underwent tendon transfer to relieve the finger drop

**Fig. 10:**
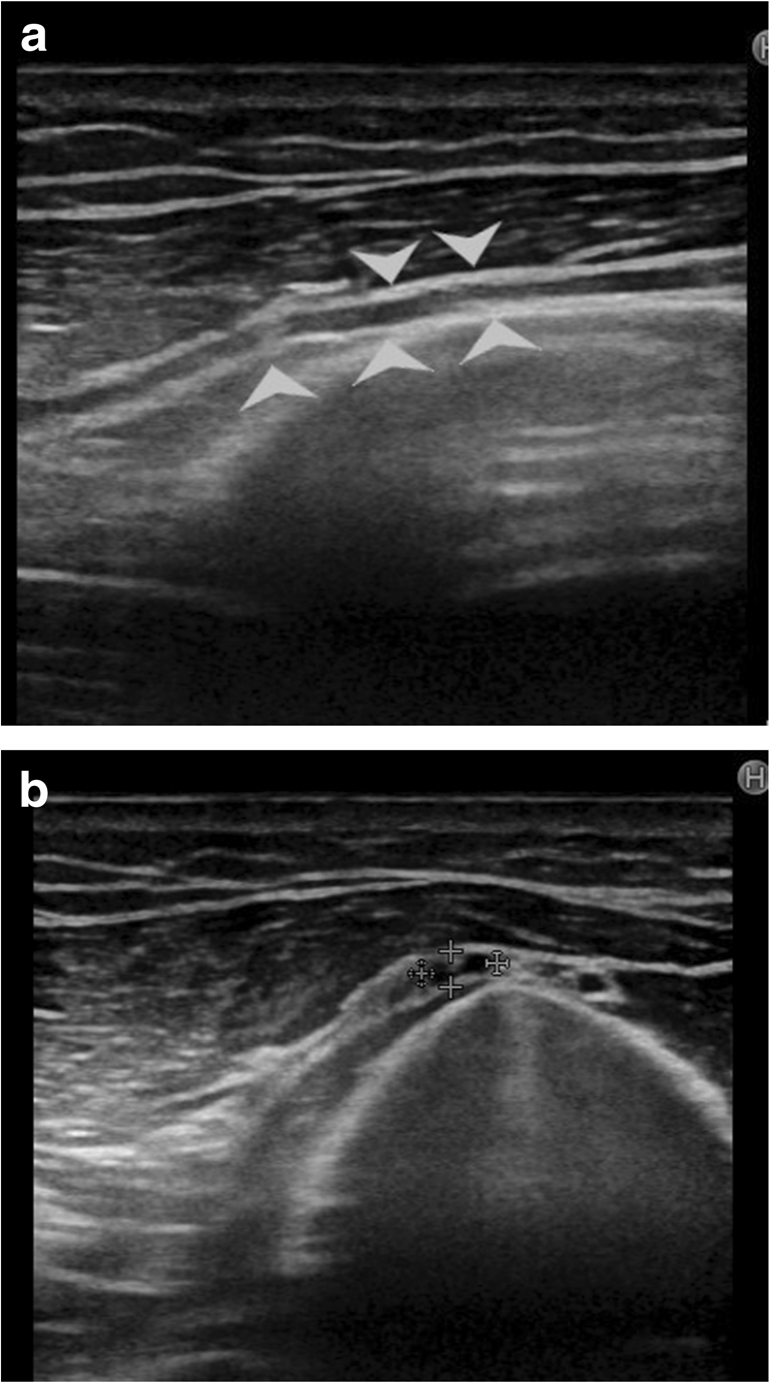
**a** Loss of normal honeycomb pattern is seen in this patient who had a tourniquet placed for forearm surgery. The patient had wrist drop in the post-operative period which has improved gradually over 3 months with physiotherapy but has not recovered completely. **b** Short axis view of radial nerve in spiral groove, between cross heads. The nerve is hypoechoic but of normal calibre

**Fig. 11:**
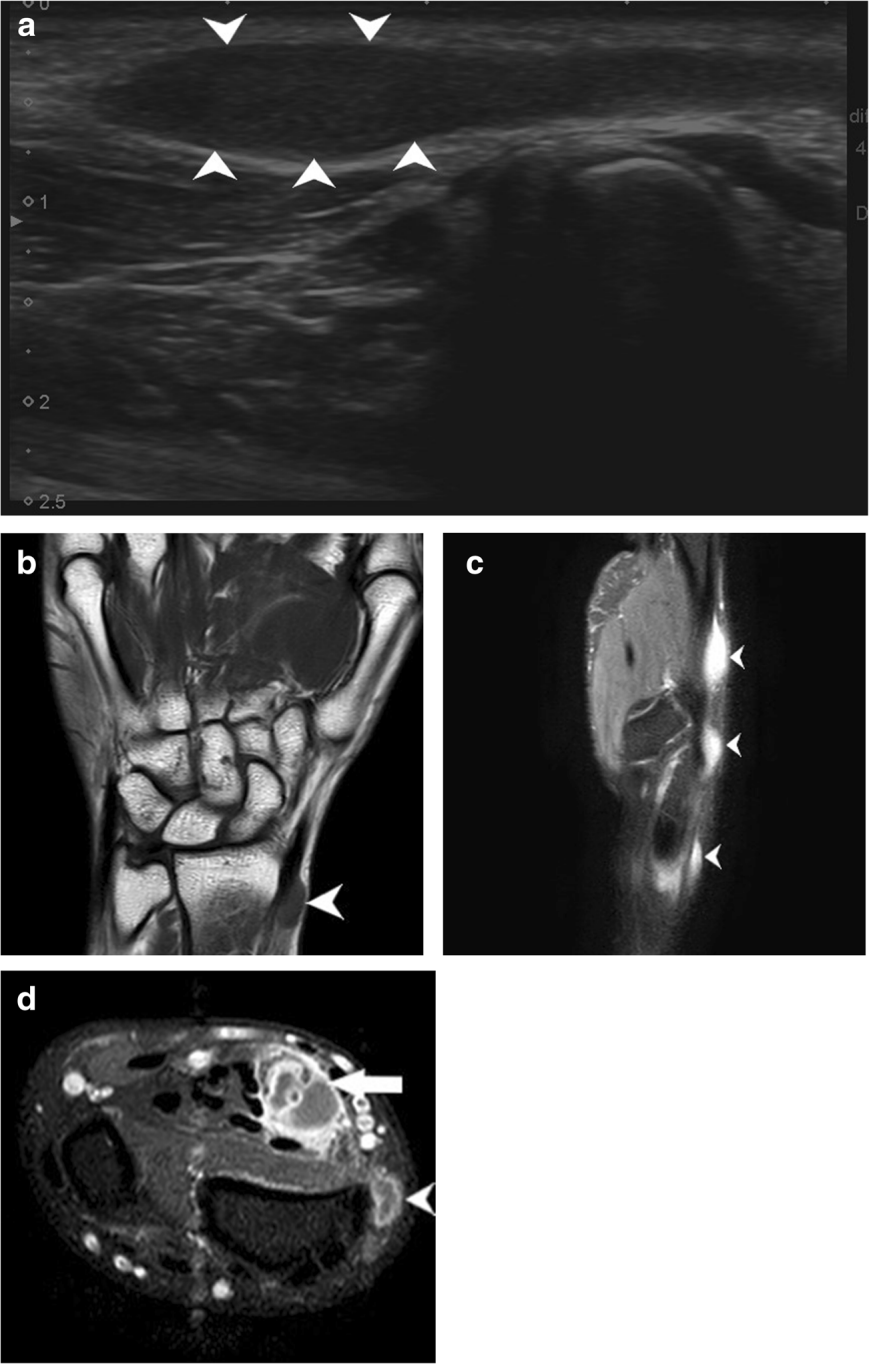
**a** Well-defined fusiform hypoechoic lesion (white arrowheads) is noted in continuity of the superficial branch of the radial nerve in the region of the anatomical snuffbox, just distal to the radial styloid. **b** T1-weighted coronal image shows another well-defined, T1 hypointense, fusiform lesion (white arrowhead) in continuity with the superficial branch of the radial nerve at the distal end of the radius. **c** PD fat-saturated sequence in the sagittal plane showing multiple PD hyperintense lesions (white arrowheads) along the superficial branch of the radial nerve. The lesions were hyperintense on T2 sequence also, showing internal fascicular areas suggestive of neural origin. **d** Axial post-contrast fat-saturated T1 image showing well-defined hypointense lesion surrounded by an enhancing capsule (arrowhead) in continuity with the superficial branch of the radial nerve. Another similar lesion (arrow) showing heterogenous contrast enhancement with hypointense areas within can be seen in continuity with the median nerve. This lesion was T2 and PD hyperintense with cystic degenerative changes on ultrasound and MRI. These MR findings fit the description of ancient schwannomas [33]. Ultrasound is a preliminary tool for identifying PNSTs, but MRI is the modality of choice as it helps in differentiating between neuromas and PNSTs

1. Post-traumatic radial nerve palsy
  Fracture of humerus shaft is the most common cause of radial nerve injury in the upper arm [23]. The overall prevalence of radial nerve palsy after fracture of the humerus shaft is 11.8% and commonly occurs following fractures of the middle and middle-distal parts of the shaft [30]. Use of high-resolution ultrasound in cases of post-traumatic radial nerve palsy helps in rapid assessment of the nerve throughout its course and helps in identifying the exact site and cause of palsy. Ultrasound is widely available, less expensive, has no contraindications and can be done at the bed side in cases of trauma. The nerve is easily identified in its short axis in the distal upper arm where it lies between the brachialis and brachioradialis and then traced proximally and distally. The pathological segment of the nerve is identified by decreased echogenicity, change in calibre, loss of continuity or impingement by or entrapment in the fracture segment.
  Figure 3 shows a hypoechoic radial nerve due to entrapment within the fracture segment in a case of road traffic accident with fracture of the humerus shaft. Management options include conservative treatment or exploration. High-resolution ultrasound can be done rapidly for such patients which allows direct visualisation of the affected segment of the nerve. Nerve entrapment in the fracture segment will require exploration, whereas local compression by hematoma can be managed conservatively and monitored for clinical improvement.
2. Penetrating trauma to the arm
  The radial nerve has a superficial course and can be injured by penetrating objects. The insult may cause complete transection of the nerve resulting in discontinuity of the nerve or result in formation of a neuroma in continuity. Such traumatic neuromas occur due to a disorganised attempt at nerve regeneration. They may be palpable as a small, firm mass or visible only on imaging as a focal fusiform mass in continuity with the nerve. As they occur due to local fibrosis, they are hypoechoic relative to surrounding muscle on ultrasound imaging [3, 29].
  Pseudo-neuroma and true neuroma can be reliably differentiated by imaging studies. Pseudo-neuromas occur due to focal enlargement of the nerve due to compression or entrapment and correspond to grade III Sunderland injury. True neuromas occur due to scarring of the nerve with fibrotic regeneration with resultant loss of fascicular architecture. This corresponds to a grade IV Sunderland type of nerve injury. Presence of fascicular architecture in an enlarged nerve on MRI signifies a pseudo-neuroma, while discontinuity of fascicular architecture is seen in a true neuroma. Figure 4 shows focal fusiform thickening of radial nerve with loss of fascicular architecture suggestive of a neuroma in continuity of the nerve in a case of wrist drop following a monkey bite. The nerve was identified by high-resolution ultrasound at the spiral groove and then followed distally. The findings of ultrasound corroborated the MRI findings.
  Figure 5 shows a case of PIN injury with discontinuity of the nerve following penetrating trauma by a pair of scissors. Hypoechoic scar tissue was noted at the site of nerve discontinuity.
3. Entrapment or compression neuropathy
  This may occur due to entrapment at normal anatomical sites which most commonly include PIN entrapment at the arcade of Frohse. Other sites include the triangular space, within the triceps brachii or the spiral groove. Compression by anomalous muscle, the accessory subscapularis-teres-latissimus, may also occur. Entrapment within scar tissue may occur following injury or chronic inflammatory states. Figure 6 shows entrapment of radial nerve in local scar tissue at the site of chronic inflammation in a case of malunited supracondylar fracture of the humerus. Figure 7 shows the PIN entrapped in fibrous scar tissue at the level of the radial neck. The nerve was normal proximally, distally and within the arcade of Frohse. The patient had a history of penetrating trauma and presented with weakness of the thumb, and finger drop with radial deviation of the wrist. Movement at the wrist was normal. The patient’s symptoms improved following surgical release of the nerve.
4. Iatrogenic causes
  Iatrogenic causes of radial nerve palsy may be manifold. Immediate post-operative wrist drop may be due to direct injury to the nerve, local hematoma causing compression, implant abutting the nerve or due to ‘tourniquet palsy’ or residual block.
  Figure 8 shows a radial nerve displaced by a hematoma in a post-operative patient who was a case of humerus fracture managed by internal fixation. The patient had wrist drop in the immediate post-operative period. High-resolution ultrasound and MR neurography showed the cause of compression which was managed conservatively.
  Figure 9 shows PIN compression by a nail used for plating both bones of the forearm in a patient with post-traumatic fracture of the radius and ulna. Tendon transfer was subsequently done for this patient.
  Tourniquet palsy is usually transient and recovers with physiotherapy and medical management [31]. High-resolution ultrasound may show normal echogenicity of the nerve or may show enlarged hypoechoic nerve fibres, as seen in Fig. 10. This difference in ultrasound findings might relate to the extent of damage to nerve fibres.
5. Peripheral nerve sheath tumour (PNST) [32]
  PNSTs include neurofibroma and schwannoma and may be benign or malignant. Deep-seated neurofibromas may have neurological symptoms, while superficial neurofibromas are usually small painless masses. Schwannomas typically present as a slow-growing, painless, soft-tissue mass located eccentrically with the parent nerve usually separable from the lesion.
  Plain radiography is usually normal in PNST. High-resolution ultrasound shows a hypoechoic fusiform mass in continuity with the parent nerve. MRI is the imaging modality of choice to differentiate between PNST and traumatic neuromas. Classical signs of PNST seen on MRI include a T1 iso-hypointense, T2 hyperintense fusiform mass in continuity with the nerve. The lesion may show low signal intensity in the centre on T2-weighted images corresponding to the higher collagen content in the centre. This is the characteristic target sign of PNST on MRI. Another sign is the split fat sign best seen on T1-weighted images wherein the hyperintense fat around the fusiform mass suggests intermuscular location of the lesion. A fascicular pattern may be detected within the mass which also supports its neurogenic origin. Figure 11 shows a case of multiple PNSTs in the superficial branch of the radial nerve.

## Management [29]

The appropriate management of radial nerve palsy depends primarily on accurate determination of its cause, severity, duration and level of involvement. Treatment of radial nerve palsy may be either operative or non-operative. Introduction of use of imaging in cases of neuropathy has ushered in an era of a multimodality approach in patient care and appropriate clinical decision making. Imaging helps to identify cases requiring operative interference like discontinuity in the nerve, neuroma formation, cases of entrapment, etc. Other cases like tourniquet palsy, compression by local hematoma, or post-operative neuritis may be managed conservatively and monitored clinically. Open radial nerve injuries that result in a loss of nerve continuity are managed surgically by primary repair, nerve grafts, or tendon transfers.

## Further advances in MRI

Diffusion tensor imaging and tractography are now being used for peripheral nerve pathologies. Jengojen et al. [34] used diffusion tensor imaging and tractography to assess acute changes in radial and median nerves following compression and demonstrated changes in radial nerve metrics. They concluded that the radial nerve is more vulnerable to compressive effects at the spiral groove which may reflect upon the pathogenesis of tourniquet palsy. Razek et al. [35] carried out a prospective study in 39 patients with mild to moderate carpal tunnel syndrome and found positive correlation between findings of diffusion tensor imaging, electrodiagnostic tests and clinical assessment.

Use of contrast in peripheral nerve pathologies helps in better characterisation of the lesions. True neuromas can be differentiated from PNST by lack of post-contrast enhancement in the former [36]. Hybrid PET-MRI is also being considered for diagnosis of tumour and tumour-like lesions of the nerve [29].

## Advantages and limitations of ultrasound

Use of high-resolution ultrasound in imaging of peripheral neuropathy is a relatively new use of this age-old modality. Literature review shows many studies and reports from around the world discovering this aspect of ultrasound [3–7, 27, 29, 37–41]. Ultrasound is a rapid, cost-effective, widely available modality with no contraindications. It allows quick assessment of the nerve throughout its length and provides unmatched data about anatomical details of the affected nerve. Technical issues like inability to image through bone, orthopaedic implants, poor resolution of deeper nerves with higher frequency transducers, suboptimal scan in patients with higher fatty tissue due to increase in depth and similar echogenicity limit its use to a certain extent. Operator dependence and need for in-depth knowledge of the anatomical details is another limiting factor.

## Conclusion

There are various underlying aetiologies for pathology of the radial nerve as described above. Patients presenting with symptoms of radial nerve palsy have been conventionally diagnosed using clinical and electrodiagnostic findings. Use of high-resolution ultrasound and MRI will usher in a new era of a multimodality approach in diagnosis and treatment of nerve pathologies. It allows direct visualisation of the nerve with every anatomical detail which will help in better clinical decision making. This pictorial review describes the hallmark of imaging findings in a wide spectrum of radial nerve pathologies.

## Abbreviations

PIN: Posterior interosseous nerve
MRI: Magnetic resonance imaging
PNST: Peripheral nerve sheath tumour
PD: Proton density
