# Correction to: A panorama of radial nerve pathologies- an imaging diagnosis: a step ahead

**DOI:** 10.1186/s13244-019-0699-5

**Published:** 2019-02-06

**Authors:** Aakanksha Agarwal, Abhishek Chandra, Usha Jaipal, Narender Saini

**Affiliations:** 1 Department of Radiodiagnosis and Modern Imaging, SMS Medical College and Attached Hospitals, Jaipur, Rajasthan India; 2 Department of Orthopaedics, SMS Medical College and Attached Hospitals, Jaipur, Rajasthan India

**Correction to: Insights Imaging**

**https://doi.org/10.1007/s13244-018-0662-x**

The original article [1] contains an omission in authorship detail; authors Aakanksha Agarwal and Abhishek Chandra are instead joint first authors.
